# Sputum smear conversion and treatment outcomes among drug-resistant pulmonary tuberculosis patients in eastern Ethiopia: A 9-years data analysis

**DOI:** 10.3389/fmed.2022.1007757

**Published:** 2022-12-01

**Authors:** Mulugeta Gamachu, Alemayehu Deressa, Abdi Birhanu, Galana Mamo Ayana, Temam Beshir Raru, Belay Negash, Bedasa Taye Merga, Addisu Alemu, Fila Ahmed, Ahmed Mohammed, Ibsa M. Abdulahi, Lemma Demissei Regassa

**Affiliations:** ^1^School of Medicine, College of Health and Medical Sciences, Haramaya University, Harar, Ethiopia; ^2^Department of Public Health, Rift Valley University, Harar, Ethiopia; ^3^School of Public Health, College of Health and Medical Sciences, Haramaya University, Harar, Ethiopia

**Keywords:** smear, sputum, Ethiopia, tuberculosis, pulmonary, treatment outcome

## Abstract

**Background:**

Drug-resistant tuberculosis (DR-TB) has become a public health problem throughout the world and about one-third of deaths were attributed to DR-TB from antimicrobial resistance which contributes to 10% of all TB deaths. Sub-Saharan Africa, particularly Ethiopia accounts for a significant number of TB cases. However, the scanty evidence on DR-TB contributing factors could affect the level of this deadly case tackling program. Therefore, this study aimed to assess the factors affecting sputum smear conversion and treatment outcomes among patients with DR-TB in Health facilities in Eastern Ethiopia.

**Methods and materials:**

A cross-sectional study design was employed from 10 October to 10 November 2021, in the health facilities providing DR-TB services in Harari Region and Dire Dawa city administration. The medical records of 273 DR-TB patients from 10 January 2013 to 27 December 2021, were reviewed using structured checklists. Data were entered into Epidata 3.1 version and exported to STATA 14 version for analysis. The outcome variables were Initial Sputum conversion (converted vs. not-converted) and treatment outcome (Unfavorable vs. Favorable). Sputum examination was performed using both Acid-fast bacillus (AFB) smear microscopy and Löwenstein–Jensen (LJ) culture technique. A binary logistic regression analysis was used to assess the association of independent variables with the first month sputum smear conversion, while a conditional logistic regression model was used to assess the association of treatment outcome with explanatory variables. The associations were reported using adjusted odds ratios (AORs) at a 95% confidence interval.

**Results:**

A total of 273 DR-TB patients were included in this study. The unfavorable DR-TB treatment outcome was significantly associated with the history of chewing khat (AOR = 4.38, 95% CI = 1.62, 11.84), having bilateral lung cavity on baseline chest X-ray (AOR = 12.08, 95% CI = 1.80, 2.57), having greater than 2+ smear result at baseline (AOR = 3.79, 95% CI = 1.35, 10.59), and poor adherence (AOR = 2.9, 95% CI = 1.28, 6.82). The sputum smear non-conversion at first month was significantly associated with being Human Immune Virus (HIV)-negative (AOR = 0.37, 0.17, 0.82), having low baseline BMI (AOR = 0.54, 95% CI = 0.29, 0.97), baseline culture > 2++ (AOR = 0.15, 95% CI = 0.05, 0.49) and having greater than 2+ sputum smear result (AOR = 0.09, 95% CI = 0.012, 0.67). Patients with normal chest X-ray at baseline had 3.8 times higher chance of sputum smear conversion on first month (AOR = 3.77, 1.11, 12.77).

**Conclusion:**

The overall initial sputum smear conversion and the treatment success rate among DR-TB patients were 52.75 and 66.30%, respectively. The Baseline underweight, HIV-negative, baseline smear > 2+, baseline culture > 2++, and clear lung on baseline X-ray were associated with smear conversion and history of khat chewing, bilateral lung cavity at baseline, having greater than 2+ smear results at baseline, and patients with poor treatment adherence had hostile treatment outcomes. So, strengthening and implementing nutrition assessment and patient counseling during directly observed therapies (DOTs) service and drug compliance could result in early sputum conversion and better treatment outcomes. DR-TB patients with high bacterial load and abnormal lungs on radiologic examination at baseline could need special attention during their course of treatment.

## Introduction

Based on global Tuberculosis (TB) report of 2019, around 3.3% of new TB cases and 17.7% of previously treated cases had multidrug-resistant TB (MDR-TB) or Rifampicin-resistant TB (RR-TB) (1, 2). In Sub-Saharan Africa, the pooled prevalence of DR-TB in new cases was 2.1% which shows a non-significant decline (3). Specifically, the overall magnitude of drug-resistant tuberculosis (DR-TB) was high in eastern Ethiopia account 3.8% (4).

Drug-resistant tuberculosis has become a public health problem throughout the world and about one-third of deaths were attributed to DR-TB from antimicrobial resistance which contributes to 10% of all TB deaths (5). Based on the global TB report, 3–4% of new and 18–21% of previously treated cases are diagnosed with MDR/RR-TB, and most cases were occurred in Asia and Africa (6). In Ethiopia RR-TB and MDR-TB is a major public health problem, affecting a productive segment of the population with an estimated prevalence of 1.1 and 1.03% among new and 7.5 and 6.52% among previously treated TB cases, respectively in 2019 (7). Moreover, DR-TB had a significant impact on the economy, and psychological and social stigma. Therefore, the problem related to DR-TB is not only affecting individual patients and their families, but also public health systems designed to prevent and control the burden of the disease (2, 8, 9).

Even though, the cost-effective directly observed therapy (DOT) strategy highly helps many world countries in controlling and preventing the disease; however, the prevalence of DR-TB has been increasing in the last 15 years, especially in poor economic countries due to a shortage of effective diagnostic and treatment access (8, 10).

For patients on treatment with confirmed pulmonary DR-TB, World Health Organization (WHO) and national TB guidelines recommend monthly sputum smear microscopy tests to predict the treatment outcome and to follow the occurrence of treatment failure (2, 9). A change in the sputum smear test from baseline positive result to negative or non-detectable result suggests a good TB treatment response. Initial or 1-month smear result is very helpful to deciding to change other alternative drugs that may result in favorable end treatment outcome. In addition, the sensitivity and specificity of sputum smear conversion to predict the treatment outcome of DR-TB patients was high compared to the sputum culture conversion (11). Recently, there are many options to diagnosis and monitoring TB treatment responses such as culture, GeneXpert (less specificity), sputum smear microscopy Mycobacterial Load Assay (MBLA) and interferon-γ release assay (12, 13).

Even though it is the gold standard, culture is not conducted in the current study area; the sputum sample is sent to the central part of the country where the culture procedure is available and it takes several weeks/month to obtain the result. Sputum smear is recommended for developing and low-resource countries due to its cost-effectiveness and less time-consuming. Therefore, there is a pressing need for evidence-based policy recommendations on the treatment and care of patients with DR-TB especially in low-income countries, based on the most recent and comprehensive evidence available (2). So, this study is aimed to assess the initial sputum smear conversion and treatment outcome among DR-TB patients on treatment in Harari regional state and Dire Dawa Administration from January 10/2013 to December 27/2021.

## Methods and materials

### Study design, period, and area

An Institutional based cross-sectional study design was conducted in Harari regional state and Dire Dawa city administration, east Ethiopia from 10 October to 10 November 2021. The Harari regional state and Dire Dawa were the most populated cities in the Eastern part of Ethiopia. The distance between them was 55 Km. The Harari region is one of the 10 regional administrations in Ethiopia and is located 522 km from Addis Ababa to the east of the country. Based on the 2007 Census conducted by the Central Statistical Agency of Ethiopia (CSA), Harari has a total population of 183, 415 (14). The Region has 3 public hospitals and 8 health centers among these Amir Nur is the only health center where DR-TB treatment is provided since 2016. Dire Dawa city administration is located at a distance of 515 km from Addis Ababa to the eastern of the country. According to the 2019/20 report of CSA the total population of Dire Dawa was 506,639 (15). The city has two public Hospitals and 15 Health centers, Dire Dawa health center is only the health center where DR-TB treatment is provided since 2013.

### Population and eligibility criteria

The source population of the study was all DR-TB patients diagnosed and recorded in the study health facilities. The study population was all DR-TB patients who fulfilled the inclusion criteria for this study. All DR-TB patients who had positive sputum smear at baseline and at least followed for 2 months before the actual data collection were included in the study retrospectively. DR-TB patients who had an incomplete measurement for the variable of interest were excluded from the study.

### Sample size and sampling procedures

The minimum required sample size for the current study was calculated using a sample size determination of single population formula using Epi-Info version 7.2.5 statistical software with consideration of the following statistical assumptions: 95% confidence interval, 5% margin of error, and after calculating for other variables related to our study, a 17.86% proportion of unfavorable treatment outcome was a variable with the largest sample size from a previous study conducted (16). After considering a 5% of withdrawal probability, the final sample was determined to be 236. However, this study was included all medical charts of patients who met inclusion criteria from both study settings. Totally, 284 patients were treated at both health centers (39 at Harari and 245 at Dire Dawa) since 2013. Depending on this, 35 charts of patients from Amir Nur center and 238 charts of patients from Dire Dawa health center were included in this study. Four charts from Amir Nur center and seven from Dire Dawa health center were excluded from the study. Therefore the final sample size for the survey was *n* = 273.

### Study variables and measurements

The two outcome variables were initial sputum smear conversion (conversion within 1 month of anti-TB initiation) and DR-TB treatment outcome (Favorable vs. Unfavorable). The independent variables included socio-demographic characteristics such as age, sex, region, residence, current marital status, educational status, and occupation; behavioral risk factors such as alcohol drinking, history of cigarette smoking, and history of chat chewing and clinical characteristics such as resistance type, regimen eligibility, Human Immune Virus (HIV) status, other Comorbidities conditions, BMI, chest radiography, ECG finding, and adherence level.

#### Operational definitions

Sputum smear conversion: is defined as a smear-positive Pulmonary-TB cases who became smear negative after a period of anti-TB treatment and it is confirmed by at least two consecutive negative sputum Acid-fast bacillus (AFB) by taking sputum smear 30 days apart. In this study, the sputum smear conversion time was measured on a monthly scale.

Drug-resistant-TB: is a form of antimicrobial resistance that is difficult and costly to treat. It is caused by TB bacteria that are resistant to at least one of the first-line existing TB medications.

Mono-resistant TB: is resistant to one first-line anti-TB drug only.

Multi-resistant TB: is resistant to at least both isoniazid and rifampicin of first-line anti-TB drugs.

#### Treatment outcomes

All outcomes were defined and classified according to the WHO guidelines (8, 16).

Cured: Treatment completion without evidence of failure and three or more consecutive negative cultures at an interval of at least 30 days after the intensive phase.

Treatment completed: Treatment completed according to national recommendation without evidence of failure but no record that three or more consecutive cultures taken at least 30 days apart are negative after the intensive phase.

Died: A patient who dies during TB treatment.

Treatment failed: Treatment terminated or need for permanent regimen change of at least two anti-TB drugs.

Lost to follow-up: A patient whose treatment was interrupted for two consecutive months or more.

Not evaluated: A TB patient for whom no treatment outcome is assigned.

Favorable (successful) treatment outcomes: include cured patients and those who completed treatment.

Unfavorable (unsuccessful) treatment outcome: include lost in follow-up, treatment failure, or death.

Adherence: is defined as taking > 90% of medications under conditions of direct observation by another person (7). The treatment adherence was measured by both subjective measurements obtained by asking patients, family members, caregivers, and physicians about the patient’s medication use and objective measurements obtained by counting pills. In this study, adherence status was judged by physicians/trained nurses and recorded on adherence sheet as good, fair and poor.

### Laboratory examination producers

#### AFB smear (microscopic examination)

All the monthly collected sputum were stained for AFB and examined using a 100 times magnified objective lens of the Ziehl–Neelsen (ZL) method. The degree of sputum AFB positivity or grading was assigned to one of the five categories (negative, scanty, 1+, 2+, and 3+). The sputum bacillary load was graded as negative when there were no tuberculosis bacilli per 100 field of observation, as scanty when there was one to nine bacilli per 100 field of observation, as plus one (1+) when there was 10–99 bacilli per 100 fields of observations, plus two (2+) when the observed bacilli were 1–10 per field, and three plus (3+) when the number of bacilli was greater than 10 per field of observation (17, 18).

#### Sputum culture procedure

The isolation of MTB in sputum cultures was performed in Löwenstein–Jensen (LJ) solid medium and recorded as follows; no growth (none), record actual number (1–9 colonies), 1+ (10–100 colonies), 2+ (> 100–200 colonies), 3+ (> 200 colonies), positive for other mycobacteria (other mycobacterial growth), contaminated (contaminated), and positive for MTB and contamination (17, 18).

#### Data collection procedure and quality control

A checklist was prepared to collect 9 years (from 10 January 2013 to 27 December 2021) of relevant data of DR-TB patients by reviewing patient charts and/or medical records. Health professionals were assigned as data collectors and data was extracted by reviewing follow-up charts and cards of patients. The 2-day training was given to the data collectors on the objective of the study and how to review the documents as per the data extraction checklist. The principal investigators supervised the data collectors closely. The suitability of the checklist was appraised and vague questions were modified before the actual data collection. Moreover, daily monitoring of data for completeness, and double data entry were performed to see consistency in the data.

### Statistical analysis

Data were cleaned, checked for consistency and completeness before the actual data entry. Data were entered using Epidata version 3.1 and exported to STATA software version 14.2 for further statistical analysis. Descriptive statistics were conducted using frequency and percentages for categorical measurements and mean and interquartile ranges were used to summarize continuous variables. For each predictor, a bi-variable binary logistic model and conditional logistic regression were fitted. In the bi-variable analysis, predictors with a *p*-value less than 0.25 were selected to be included. Multivariable logistic regression was used to assess the association of independent variables in the first month of sputum smear conversion. The conditional logistic regression model was used to assess the association of treatment outcome and independent variables using initial sputum smear conversion (converted vs. not converted) as group variables. The association between outcomes and explanatory variables was reported by adjusted odds ratios (AORs) with a 95% confidence interval. A statistically significant association between the outcome variables and predictors was detected by using a 95% confidence interval. Multicollinearity assumptions were checked through the variance inflation factor (VIF) where non-collinear means the VIF of all variables should be less than 10. Moreover, after fitting the model, the goodness of the final model was checked by using the Hosmer–Lemeshow test. The Hosmer–Lemeshow statistic indicates a good fit at the *p*-value of 0.05 or greater. Finally, the significance level was declared at a *P*-value less than 0.05.

## Results

### Socio-demographic and behavioral characteristics

A total of 284 bacteriologically confirmed DR-TB patients started RR/MDR-TB treatment regimens from 10 January 2013 to 27 December 2021. Eleven patients who had a negative sputum smear at baseline were excluded from the study. The data of 273 patients were included in the final analysis.

The mean [±standard deviation (SD)] age of the study participants was 31 (SD ± 13.7) years with a range of 6–76 years. The majority of participants were male 171 (62.24%); more than half 144 (52.75%) of the study participants were from Dire Dawa. Around half, 128 (46.89%) patients were referred from General hospitals. The majority, 236 (86.4%), of patients were from urban residences. Ninety-two (33.70%) participants were single and 47 (17.22%) patients were students. Regarding the behavioral characteristics of respondents, 67 (24.54%), 89 (32.60%), and 108 (39.56%) were having a history of alcohol drinking, cigarette smoking, and chat chewing at baseline, respectively (Table 1).

**TABLE 1:** Socio-demographic and behavioral characteristics of DR-TB patients in Harari regional state and Dire Dawa city administration, eastern Ethiopia, from 2013 to 2021 (*N* = 273).

Variables *n* = 273	Categories	Frequency	Percent (%)
Age category	≤ 14 years	16	5.86
	15–30 years	147	53.85
	31–45 years	64	23.44
	46–60 years	40	14.65
	> 60 years	6	2.20
Sex	Male	171	62.24
	Female	102	37.36
Region of patients	Harari	32	11.72
	Dire Dawa	144	52.75
	Oromia	25	9.16
	Somali	68	24.91
	Others*	4	1.47
Residence	Urban	236	86.45
	Rural	37	13.55
Current marital status	Single	92	33.70
	Married	44	16.12
	Divorced	9	3.30
	Widowed	2	0.73
	Not documented	126	46.15
Educational status	No formal education	17	6.23
	Primary (1–8)	44	16.12
	Secondary (9–12)	47	17.22
	Above	18	6.59
	Unknown	147	53.85
Occupation	Government employee	20	7.33
	Self-employee	32	11.72
	Student	47	17.22
	Farmer	20	7.33
	Others**	16	5.6
	Not documented	138	50.4
Referring health facilities	Comprehensive Specialized hospital	23	8.42
	General Hospital	128	46.89
	Primary hospital	24	8.79
	Health center	98	35.90
Year of registration	2013	40	14.65
	2014	32	11.72
	2015	26	9.52
	2016	27	9.89
	2017	43	15.75
	2018	13	4.76
	2019	27	9.89
	2020	30	10.99
	2021	35	12.82
History alcohol drinking	No	206	75.46
	Yes	67	24.54
History of cigarette smoking	No	184	67.40
	Yes	89	32.60
History of chat chewing	No	165	60.44
	Yes	108	39.56

*Addis Abeba and Amhara, **drivers and street children’s.

### Clinical characteristics

Out of the 273 patients with initial smear positive, 58 (21.25%) patients were new cases and 97 (34.43%) were on retreatment after the first-line anti-TB regimen failure. Regarding the type of drug resistance, 41 (15.02%) of the patients were resistant to both isoniazid (INH) and rifampicin (RIF), while 232 (84.98%) were resistant to only rifampicin (RIF). The majority of patients, 204 (74.73%), were treated with a standardized long DR-TB regimen. Fifty-one (18.68%) of study participants were HIV positive, while 24 (10.81%) of the study participants were living with other comorbidities. The commonest comorbidity was diabetes mellitus (DM), 14 (5.13%). Nearly two-thirds, 178 (65.20%), had a BMI < 18.5 kg/m^2^; 186 (68.13%) of patients had good adherence to treatment. Regarding the end of treatment outcomes of patients, 115 (42.12%) were cured; and 30 (10.99%) died during the treatment period (Table 2).

**TABLE 2:** Baseline clinical characteristics of DR-TB patients in Harari regional state and Dire Dawa city administration, eastern Ethiopia, from 2013 to 2021 (*N* = 273).

Variables *n* = 273	Categories	Frequency	Percent (%)
Registration group	New	58	21.25
	Relapse	71	26.06
	After the failure of the new FLD	37	13.55
	After the failure of treatment FLD	97	34.43
	Transfer in	13	4.76
Resistance type	Rifampicin resistant TB	232	84.98
	Multidrug-resistant TB	41	15.02
Regimen eligibility	Standardized Long DR-TB treatment regimen	204	74.73
	Individualize long DR-TB treatment regimen	43	15.75
	Short DR-TB treatment regimen	26	9.52
HIV status	Positive	51	18.68
	Negative	222	81.32
Comorbidity conditions other than HIV	Yes	24	10.81
	No	198	89.19
Baseline BMI categories	< 18.5 kg/m^2^	178	65.20
	≥ 18.5 kg/m^2^	95	34.80
Baseline chest radiography	Unilateral lung cavity	28	10.26
	Bilateral chest cavity	23	8.42
	Abnormal without cavity	172	63.00
	Clear lung	37	13.55
	Unknown	13	4.76
Baseline ECG result	Normal	156	57.14
	Abnormal	6	3.30
	Not done	108	39.56
Status of adherence to treatment	Good	186	68.13
	Fair	54	19.78
	Poor	33	12.09
Outcome of patients	Cured	115	42.12
	Completed	66	24.18
	Died	30	10.99
	LFU	24	8.79
	Not evaluated	38	13.92

FLD, first-line drugs; TB, tuberculosis; DR-TB, drug-resistant tuberculosis; LFU, lost to follow-up.

### Microbiological and laboratory test results

The majority, 219 (80.22%), of study participants had 1+ baseline sputum smear results; 178 (65.20%) were had 1+ culture results at the baseline; 240 (87.91%), and 245 (94.25%) had normal WBC (4,500–11,000/mm^3^) and RBC (4.0–5.6 million/mm^3^) at the baseline, respectively. Thirteen (5%) patients had low (< 3.5 mmol/L) K + levels; 12 (4.39%) patients had low (< 136 mmol/L) N + levels, and 17 (6.22%) participants had high baseline creatinine levels (Table 3).

**TABLE 3:** Baseline microbiological and laboratory test results of DR-TB patients in Harari regional state and Dire Dawa city administration, east Ethiopia, from 2013 to 2021 (*N* = 273).

Variables (*n* = 273)	Categories	Frequency	Percent (%)
Baseline smear result	< 1+	219	80.22
	> 2+	54	19.78
Baseline culture result	Actual count (1–9)	66	24.18
	10–99 (1+)	178	65.20
	> 100 (2++)	29	10.62
Baseline WBC result	High (> 11,000/mm^3^)	13	5.00
	Normal (4,500–11,000/mm^3^)	245	94.23
	Low (< 4,500/mm^3^)	2	
	Not done	13	4.76
Baseline RBC result	High (> 5.6 million/mm^3^)	3	
	Normal (4.0–5.6 million/mm^3^)	240	87.91
	Low (< 4.0 million/mm^3^)	14	5.12
	Not done	16	5.86
Baseline K+ level (mmol/L)	Normal (3.5–5 mmol/L)	116	42.49
	Low (< 3.5 mmol/L)	13	5.00
	High (> 5 mmol/L)	11	4.02
	Not done	133	48.71
Baseline Na+ level (mmol/L)	Normal (136–146 mmol/L)	76	27.83
	Low (< 136 mmol/L)	12	4.39
	High (> 146 mmol/L)	8	2.93
	Not done	177	64.83
Baseline Creatinine	High (> 1.5 mg/dL)	17	6.22
	Normal (0.7–1.5 mg/dL)	220	80.58
	Low (< 0.7 mg/dL)	3	
	Not done	33	12.08

### Initial sputum smear conversion and associated factors among drug-resistant tuberculosi patients

Overall, 144 (52.75%) patients converted their sputum smear on the first month of treatment initiation and 129 (47.25%) patients’ sputum smear did not convert.

Patient’s characteristics such as residence, history of cigarette and khat use, type of resistance, registration group, baseline BMI, HIV/IADS status, baseline chest X-ray results, baseline line smear result, baseline culture result, and status of drug adherence had a *p* < 0.25 in bi-variable regression analysis and included in the final multivariable analysis.

This study found that DR-TB patients with baseline underweight, HIV-negative, baseline smear > 2+, baseline culture > 2++ were significantly associated with initial sputum non-conversion, and patients with clear lung X-ray on baseline were significantly associated with initial sputum smear conversion. The study reveals that the odds of having initial sputum smear conversion among underweight patients decreased by 46% compared to normal body weight patients. DR-TB patients who were HIV-negative were 43% less likely to have first month smear conversion compared with HIV-positive patients. In addition, this study found that the odds of initial sputum smear conversion in patients with greater than 2+ sputum smear results and greater than 2++ culture results at baseline decreased by 91 and 85%, respectively. Furthermore, the current study revealed that patients with clear lung X-ray on baseline had a 3.77 times higher chances of sputum smear conversion in the first month than patients with bilateral lung cavities at baseline (Table 4).

**TABLE 4:** Factors associated with first month sputum smear conversion among DR-TB patients in eastern, Ethiopia from 2013 to 2021 (*N* = 273).

Variable (*n* = 273)	Categories	COR (95% CI)	AOR (95% CI) and *P*-value	
			AOR (95% CI)	*P*-value
Residence	Urban	1	1	
	Rural	0.64 [0.31, 1.29]	0.50 [0.22, 1.16]	0.12
History of khat	No		1	
	Yes	0.61 [0.37, 0.99]	0.51 [0.22, 1.20]	0.125
History of smoking	No	1	1	
	Yes	0.67 [0.40, 1.11]	1.27 [0.53, 3.06]	0.58
Registration group	New	1	1	
	Relapse	0.84 [0.42, 1.69]	1.22 [0.54, 2.75]	0.626
	After the failure of the new FLD	1.27 [0.55, 2.94]	1.73 [0.64, 4.65]	0.281
	After the failure of Rx. FLD	1.12 [0.58, 2.17]	1.32 [0.61, 2.84]	0.478
	Transfer in	0.26 [0.06, 1.04]	0.40 [0.08, 1.87]	0.247
Baseline BMI	≥ 18.5 kg/m^2^	1	1	
	< 18.5 kg/m^2^	0.63 [0.38, 1.05]	0.54 [0.29, 0.97]	0.038
HIV co-infection status	Positive	1	1	
	Negative	0.60 [0.32, 1.13]	0.37 [0.17, 0.82]	0.014
Baseline chest X-ray result	Unilateral lung cavity	1	1	
	Bilateral lung cavity	0.96 [0.25, 3.72]	2.38 [0.48, 11.76]	0.288
	Abnormal without cavity	1.90 [0.84, 4.31]	2.04 [0.73, 5.64]	0.170
	Clear lung	3.21 [1.15, 8.96]	3.77 [1.11, 12.77]	0.033
	Others*	0.82 [0.26, 2.59]	0.60 [0.151, 2.45]	0.486
Baseline smear result	< 1+	1	1	
	> 2+	0.56 [0.29, 0.87]	0.09 [0.012, 0.67]	0.018
Baseline culture result	Actual count (1–9)	1	1	
	1+	0.57 [0.31, 1.02]	0.56 [0.28, 1.094]	0.089
	2++	0.24 [0.09, 0.61]	0.15 [0.05, 0.49]	0.001
Drug adherence	Good	1	1	
	Fair	0.95 [0.52, 1.75]	0.89 [0.44, 1.78]	0.736
	Poor	0.53 [0.25, 1.13]	0.41 [0.15, 1.11]	0.080
Treatment outcome	Favorable	1	1	
	Unfavorable	0.56 [0.34, 0.94]	0.69 [0.35, 1.38	0.29

*Unspecified findings.

### Treatment outcome and associated factors among drug-resistant tuberculosi patients

Of the 273 DR-TB patients included in the study, the majority 115 (42.12%) were cured, followed by completed 66 (24.15%). The overall treatment success rate (cured and completed) was 181/273 (66.30%). Thirty (10.99%) treated patients died and 24 (8.79%) patients were recorded as lost to follow-up (LFU). Of the 144 patients who had converted sputum smear in the first month, only 37 (25.69%) patients had unfavorable treatment outcomes while 55 patients (42.65%) among 129 patients who did not have sputum smear conversion in the first month had unfavorable treatment outcome (Table 5).

**TABLE 5:** Treatment outcomes of DR-TB patients, eastern, Ethiopia from 2013 to 2021 (*N* = 273).

DR-TB treatment outcome (*n* = 273)		Initial conversion		Frequency (%)
		Yes	No	
Favorable *N* = 181 (66.30%)	Cured	71	44	115 (42.12)
	Completed	36	30	66 (24.18)
Unfavorable *N* = 92 (33.70%)	Died	11	19	30 (10.99)
	LFU	8	16	24 (8.79)
	Not evaluated	18	20	38 (13.92)
Total		144	129	273 (100%)

LFU, lost to follow-up.

The multivariable conditional logistic regression model indicated that history of khat chewing, bilateral lung cavities at baseline, sputum smear > 2+ at baseline, and poor treatment adherence were significantly associated with unfavorable treatment outcomes. For patients who did not have sputum smear conversion in the first month; having a history of chewing khat increases the odds of unfavorable treatment outcomes in DR-TB patients by four folds (AOR = 4.38, 95% CI = 1.62, 11.84), patients with bilateral lung cavities on the baseline were 12.08 times more likely to have unfavorable DR-TB treatment outcome (AOR = 12.08, 95% CI = 1.80, 2.57). Furthermore, DR-TB patients having greater than 2+ sputum smear results at baseline had 3.8 times odds of the unfavorable treatment outcome (AOR = 3.79, 95% CI = 1.35, 10.59), and patients who poorly adhered to the treatment 2.9 times more likely to have unfavorable treatment outcome (AOR = 2.9, 95 CI = 1.28, 6.82) (Table 6).

**TABLE 6:** Factors associated with treatment outcome among DR-TB patients eastern, Ethiopia 2022.

Variables	Categories	COR (95% CI)	AOR (95% CI) and *P*-value	
			AOR (95% CI)	*P*-value
Residence	Urban	1	1	
	Rural	0.93 [0.44, 1.96]	0.47 [0.16, 1.39]	0.172
History of khat	No	11	1	
	Yes	2.23 [1.33, 3.74]	4.38 [1.62, 11.84]	0.004
History of smoking	No	1	1	
	Yes	1.47 [0.86, 2.51]	0.61 [0.27, 1.76]	0.356
Marital status	Single	1	1	
	Married	1.90 [0.90, 4.01]	1.72 [0.56, 5.44]	0.33
	Divorced	3.94 [0.91, 17.04]	3.9 [0.44, 0.3645]	0.22
	Widowed	1.65 [0.09, 27.62]	4.40 [0.20, 97.96]	0.35
	Not known	0.84 [0.46, 1.53]	0.39 [0.14, 1.09]	0.07
Registration group	New	1	1	
	Relapse	1.45 [0.71, 2.93]	1.68 [0.69, 4.09]	0.25
	After the failure of the new FLD	0.50 [0.20, 1.25]	0.38 [0.12, 1.35]	0.13
	After failure FLD treatment	0.38 [0.18, 0.80]	0.44 [0.17, 1.14]	0.09
	Transfer in	1.12 [0.33, 3.80]	1.34 [0.29, 6.19]	0.70
Baseline BMI	≥ 18.5 kg/m^2^	1	1	
	< 18.5 kg/m^2^	1.08 [0.63, 1.86]	1.49 [0.69, 3.19]	0.30
HIV co-infection status	Positive	1	1	
	Negative	0.45 [0.24, 0.85]	0.68 [0.29, 0.57]	0.37
Baseline chest X-ray result	Unilateral lung cavity	1	1	
	Bilateral lung cavity	4.50 [1.4, 10.19]	12.08 [1.80, 2.57]	0.01
	Abnormal without cavity	0.47 [0.20, 1.08]	0.77 [0.23, 2.56]	0.67
	Clear lung	1.87 [0.68, 5.15]	3.52 [0.90, 13.68]	0.07
	Unknown	0.68 [0.21, 2.16]	1.48 [0.32, 6.90]	0.61
Baseline smear result	< 1+	1	1	
	> 2+	1.89 [0.98, 3.67]	3.79 [1.35, 10.59]	0.01
Baseline culture result	Actual count (1–9)	1	1	
	1+	0.85 [0.46, 1.56]	0.59 [0.27, 1.32]	0.20
	2++	0.80 [0.31, 2.06]	0.51 [0.14, 1.95]	0.33
Drug adherence	Good	1	1	
	Fair	1.59 [0.83, 3.06]	2.4 [0.09,7.05]	0.08
	Poor	1.89 [4.10, 9.25]	2.9 [1.28, 6.82]	0.01

## Discussion

In this study, the overall sputum smear conversion at 1 month was observed in 144 (52.75%) bacteriologically confirmed DR-TB patients, while 129 (47.25) of the patients had non-converted sputum. Being underweight at baseline, HIV negative, having greater than 2+ sputum smear result grades at baseline and normal lung x-ray at baseline were significantly associated with initial sputum smear conversion. The overall favorable treatment outcome was 66.30%. This is comparable with the study conducted in Ethiopia (19). The study revealed that Khat chewing, baseline smear result greater than 2+, poor anti-TB adherence and bilateral lung cavity had a significant association with unfavorable DR-TB treatment outcome.

The odds of initial sputum smear conversion among underweight DR-TB patients at the baseline decreased by 46% compared to patients with normal BMI. This finding is supported by the report from Ethiopia (20–22), India (23), (24), and Indonesia (25). It’s a fact that having a normal body mass index is a sign of relative normal physiological function. Low BMI (< 18.5 kg/M^2^) is an indicator of poor nutritional status or under nutrition. Malnutrition has known effects on altering the body system’s immune function, and the accompanying reduced immunity increases the susceptibility to various infectious diseases (26, 27). Insufficient protein and caloric intake can impair the function of host defense mechanisms that are essential for recovering from TB disease. Therefore, malnutrition may increase the severity of infections and decrease medications’ effectiveness due to mal absorption and impaired metabolism. These may increase the duration of sputum smear conversion and the overall recovery from the disease.

The current study revealed that patients who had normal lung radiologic finding increased the likelihood of having initial sputum smear conversion compared with those who had bilateral lung cavities. This report is in line with finding from Ethiopia (28, 29), South Africa (30), Pakistan (31), Taiwan (32), Thailand (33), and different kinds of literature (34). This could be due to the fact that lung cavitation might hinder drug penetration and thus decrease the therapeutic efficacy of the anti-TB medications and may prolong the sputum smear conversion time. Moreover, as the lung is superinfected by other infections; the time to sputum smear conversion might be prolonged due to the synergistic impact of the infections including mycobacterium tuberculosis. This probably implies that the radiological signs of the lung could offer good indicators of predicting pulmonary DR-TB treatment response. Thus, among drug resistant tuberculosis, the worse clinical manifestation including abnormal lung findings are important treatment outcome markers (35).

Patients with greater than 2+ smear result grades had longer sputum conversion times. This finding is aligned with the report from Ethiopia (21, 29), South Africa (30), Cameroon (36), Morocco (37), and Iran (38). Naturally, having high bacillary load on bacteriological examination indicates that the strength of infectivity that require special attention during treatment. The other additional explanation could be having a high load of bacilli may result in reduction of bacterial killing by the anti-TB medications. Thus, it is fact that the clearance of high bacillary load by antibiotics may take a longer time than low bacillary load.

On other hand, this study revealed that DR-TB patients with a HIV positive had a shorter time of sputum smear conversion. This is supported by a study done in Northern Ethiopia (22) and Peru (39). A contrary study conducted in Tanzania (40), showed that there was no significant difference in sputum smear conversion time between HIV-positive and negative individuals. This could be due to the variation in the level of follow-up and screening for TB-HIV co-infected hereby the co-infected patients get screened as TB/HIV integrated programs encourage early screening than those who are not co-infected. Thus, giving attention through close monitoring of TB patients is advisable. In addition, the discrepancy could be related to the time of ART initiation for HIV- positive patients; in this study almost all patients (98%) started HIV treatment before diagnosis and treatment of drug-resistant TB.

Regarding the treatment outcome, the patients who chewed khat (Catha edulis) had higher odds of having unfavorable TB treatment outcomes. This report is in line with finding from North Ethiopia (41) and Yemen (42). Chat chewing has the effect of loss of appetite which may result in immunosuppression and could also result in a functional mood disorder that may affect the medication adherence level while taking the Khat (43). The functional disorders during khat chewing are probably the reason for forgetting of the medication or discontinue the medication due to the stimulant effect of the Khat that the patient feels relief despite the pain exists. Thus, due to these all synergetic effect of Khat chewing unsuccessful treatment outcome may observed.

Patients with lung problems had unfavorable MDR TB treatment outcomes. This finding is supported by the finding from Yemen (44), China (45) and Russia (46). This can be attributed to lung cavitation is associated with higher sputum mycobacterial load at baseline, and this can be related to delay sputum smear conversion, treatment failure and death (47). It also known that when the lung tissues are damaged by tuberculosis and other superinfecting infections the probability of success rate could decrease due to overall reduction of body defense mechanism. In general, lung cavitation is a dangerous consequence of pulmonary tuberculosis associated with poor outcomes, treatment relapse, and higher transmission rates (48).

Furthermore, having poor anti-TB treatment adherence 2.8 times increases the odds of unfavorable treatment outcomes compared to those who had a good anti-TB treatment adherence. This finding agrees with the result from Ethiopia (49). This could be due to drug overload and high bacteriological load among DR-TB patients. This could also be probably forgetting to take medication, being away from home, having drug side effects or being unable to go to the health facilities on the date of appointment. Factors such as drug reactions, drug-induced gastrointestinal disorders, and psychiatric and ototoxicity-related drug side effects could make the patient non-adherent (50). Thus, if patients do not follow the required course of treatment according to the prescribed dosage, time, frequency, and direction they may remain infectious for longer and less likely to cure from the disease.

### Strength and limitation of the study

The strengths of this study were using data from two different settings and using all available patients’ medical information for 9 years of data. But the study has some limitations; use of secondary data by itself may result in inadequate access of independent factors for both sputum smear conversion and treatment outcomes.

## Conclusion

The overall initial sputum smear conversion among DR-TB patients was 52.75%. Baseline underweight, HIV-negative, baseline smear > 2+, baseline culture > 2++, and clear lung on baseline X-ray were associated with smear conversion at 1 month of treatment initiation. Furthermore, this study found that the overall treatment success rate (cured and completed) was 66.30%. History of khat chewing, bilateral lung cavity at baseline, having greater than 2+ smear results at baseline, and patients with poor treatment adherence had unfavorable treatment outcomes. Strengthening and implementing nutrition assessment and patient counseling during DOTs strategies on their behavior and drug compliance may result in early sputum conversion and better treatment outcomes. In addition, DR-TB patients with high bacterial load and abnormal lungs on radiologic examination at baseline need special attention during their course of treatment.

## Data availability statement

The data sets used for this study are available from the corresponding author on reasonable request.

## Ethics statement

The Institutional Health Research Ethics Review Committee (IHRERC) of Haramaya University’s College of Health and Medical Sciences provided ethical approval (IHREC/176/2021). Throughout the data collection and study period, all medical record data anonymous was maintained.

## Author contributions

MG conceived the idea. All authors made a significant contribution to the work reported, whether that is in the conception, study design, execution, acquisition of data, analysis, and interpretation, or in all these areas, took part in drafting, revising, or critically reviewing the article, gave final approval of the version to be published, have agreed on the journal to which the article has been submitted, and agreed to be accountable for all aspects of the work.

## Abbreviations

DR-TB: drug-resistant tuberculosis
FLD: first-line drugs
FMOH: Federal Minister of Health
HIV: Human Immune Virus
HR: Hazard Rate
IQR: Inter Quartile Range
MTB: mycobacterium tuberculosis
MDR-TB: multi-drug-resistant tuberculosis
PTB: pulmonary tuberculosis
RR-TB: Rifampicin resistance tuberculosis
SLD: Second Line Drugs
TB: Tuberculosis
WHO: World Health Organization.
